# Improving training of laparoscopic tissue manipulation skills using various visual force feedback types

**DOI:** 10.1007/s00464-016-4972-0

**Published:** 2016-05-18

**Authors:** Daan Smit, Edward Spruit, Jenny Dankelman, Gabrielle Tuijthof, Jaap Hamming, Tim Horeman

**Affiliations:** 1 Cognitive Psychology, Institute of Psychology, Leiden University, Leiden, The Netherlands; 2 BioMechanical Engineering, Delft University of Technology, Mekelweg 2, 2628 CD Delft, The Netherlands; 3 Department of Orthopedic Surgery, Academic Medical Center, Amsterdam, The Netherlands; 4 Department of Surgery, Leiden University Medical Center, Leiden, The Netherlands

**Keywords:** Visual feedback, Learning curve, Laparoscopy, Tissue manipulation skills, Force, Hybrid box trainer

## Abstract

**Background:**

Visual force feedback allows trainees to learn laparoscopic tissue manipulation skills. The aim of this experimental study was to find the most efficient visual force feedback method to acquire these skills. Retention and transfer validity to an untrained task were assessed.

**Methods:**

Medical students without prior experience in laparoscopy were randomized in three groups: Constant Force Feedback (CFF) (*N* = 17), Bandwidth Force Feedback (BFF) (*N* = 16) and Fade-in Force Feedback (*N* = 18). All participants performed a pretest, training, post-test and follow-up test. The study involved two dissimilar tissue manipulation tasks, one for training and one to assess transferability. Participants performed six trials of the training task. A force platform was used to record several force parameters.

**Results:**

A paired-sample *t* test showed overall lower force parameter outcomes in the post-test compared to the pretest (*p* < .001). A week later, the force parameter outcomes were still significantly lower than found in the pretest (*p* < .005). Participants also performed the transfer task in the post-test (*p* < .02) and follow-up (*p* < .05) test with lower force parameter outcomes compared to the pretest. A one-way MANOVA indicated that in the post-test the CFF group applied 50 % less Mean Absolute Nonzero Force (*p* = .005) than the BFF group.

**Conclusion:**

All visual force feedback methods showed to be effective in decreasing tissue manipulation force as no major differences were found between groups in the post and follow-up trials. The BFF method is preferred for it respects individual progress and minimizes distraction.

Although laparoscopic surgery brings many advantages for patients (smaller scars and shorter hospitalization), the disadvantages are to the extent of surgeons as the task complexity increases. Laparoscopic surgery requires more of the capabilities of surgeons compared to open surgery [1, 2]. Tactile feedback is degraded as a consequence of instrument friction [2–4] and between instrument and trocar [5]. Psychomotor challenges, such as counter-intuitive movement (fulcrum effect) of the instruments [3, 4] and limited degrees of motion freedom [1, 6], contribute to the increased complexity of the operating technique as well. Even though safe tissue handling is an important topic in the training of surgical skills, it is difficult to assess. New training and assessment methods were developed and validated [7] and used to provide a more objective measure for the “instrument handling” and “tissue manipulation” grading sections as used in the OSATS scoring form [8].

Previous research shows that visual force feedback contributes to safe tissue manipulation [9, 10]. However, providing frequent or continuous presentation of visual feedback does not consistently contribute to the learning process, and in some cases it may even hinder skill acquisition [11–14]. High-frequency feedback guides the trainee to correct movement [15], but overexposure can create feedback dependency (guidance effect) [11–14]. This can lead to fluctuation in performance because the trainee is constantly correcting small, insignificant errors [15].

An obvious solution to overcome the guidance effect is to omit continuous feedback [11]. This will strengthen the intrinsic ability to discriminate between skill effective and ineffective behaviour and decrease dependency on feedback [11, 14]. In this study, we aim to apply this theory by evaluating two different methods of lower-frequency feedback for laparoscopic skills training in box simulators.

## Fade-in feedback

In the literature, a number of options are suggested. One of those options to solve the guidance effect is fade-in feedback [2, 13, 16]. Feedback can possibly be overwhelming for the performer at the start of training [2] if it exceeds the attention capacity at the beginning of the acquisition process. The trainee therefore should only be presented with feedback when the surgical task demands less conscious attention of the performer (when the task has become automated).

## Bandwidth feedback

Another proposed option to undermine the guidance effect is bandwidth feedback. In this setting, the trainee will only be presented with feedback when his or her performance exceeds a certain threshold [17] and thus respects individual progress [16]. Of major importance is establishing the threshold, the tolerable amount of error before confronting the trainee with feedback. Adverse thresholds will result in overexposure (i.e. results in unstable set of execution skills) or underexposure (i.e. results in skill execution which contains errors) to augmented feedback and may lead to suboptimal performance [16].

The aim of the current study is to determine the most efficient dosage of visual force feedback using Constant Force Feedback, Fade-in Force Feedback and Bandwidth Force Feedback.

## Method

### Participants

Medical students without prior experience in laparoscopy training were recruited for the study. The study included 51 participants (30 women; mean age 19.69, range 17–30) of which 1 participant did not turn up for the follow-up test. Participants were assigned semi-randomly to one of the three groups, based on their availability. Furthermore, it was unknown for the participants that each timeslot available for training had a predefined group protocol assigned to it. The Constant Force Feedback (CFF) group consisted of 17 participants (11 women; mean age 20.12, range 18–24), the Bandwidth Force Feedback (BFF) group consisted of 16 participants (10 women; mean age 19.63, range 17–30) and the Fade-in Force Feedback (FFF) group consisted of 18 participants (9 women; mean age 19.33, range 17–28).

### Test set up

The ForMoST hybrid trainer is equipped with the TrEndo tracking system, the ForceTRAP force tracking system and an USB camera for the visualization of the task on the computer screen [18]. The ForMoST system measures all instrument movement and forces exerted on the training task.

### Tasks

To assess the surgical skills required for proper tissue handling, two tasks validated for force parameters were used [7, 10], which make use of elastic elements that mimic properties of real tissue. Bimanual cooperation of the instruments is essential to complete both tasks.

#### Task 1

**Fig. 1:**
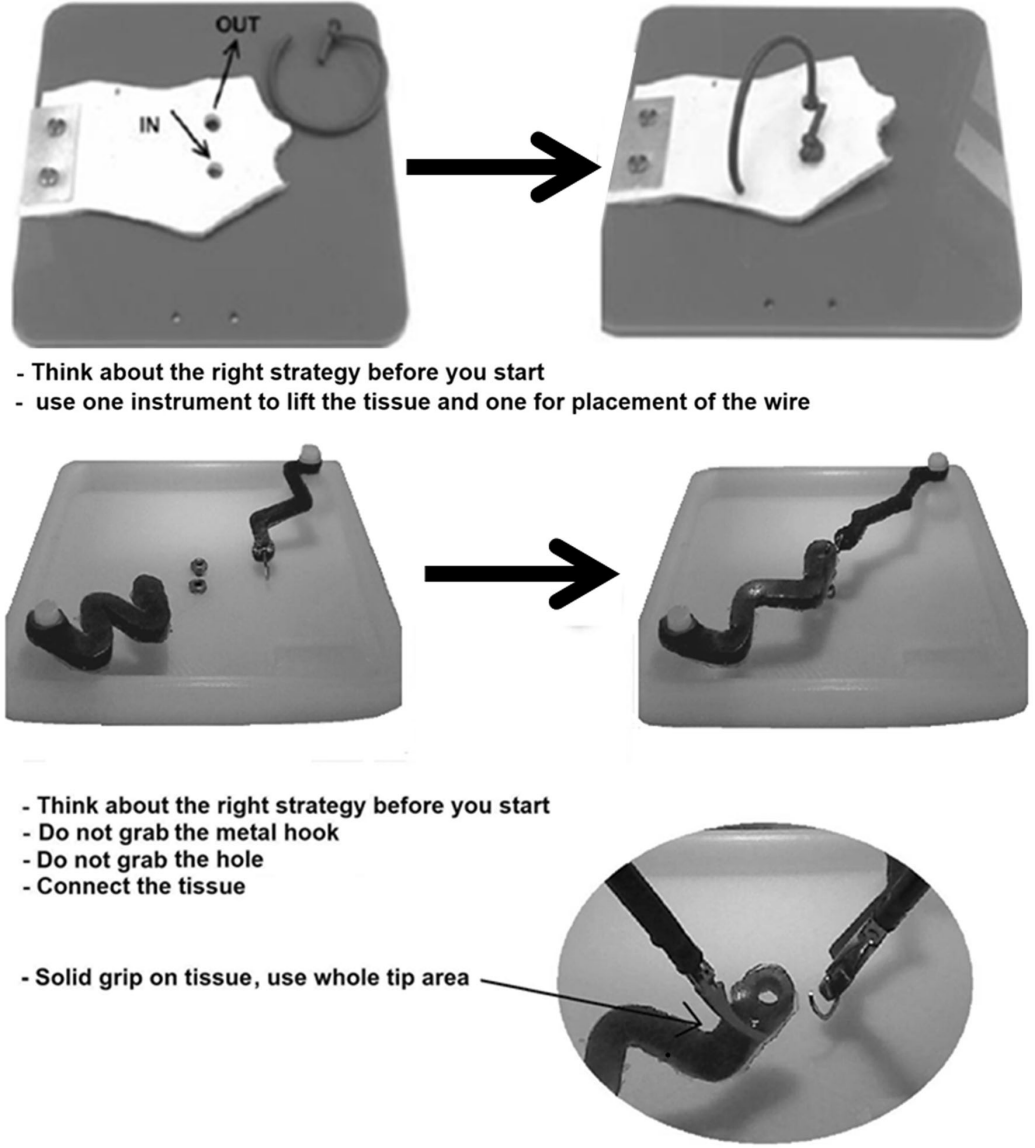
Instructions for Task 1 (*top*) and Task 2 (*bottom*) as they were presented onto the display of the hybrid trainer

The objective of the task was to guide the wire completely through the two holes of the patch, using a predefined route [7, 10] (Fig. 1). The task is designed to force participants to work bimanually with both instruments. If Task 1 is performed correctly, the applied force is negligible.

#### Task 2

In order to complete Task 2 successfully, connection of the silicon strips should be accomplished with insignificant exerted force. Different from the original task as described in our previous work [7, 10], the two silicon strips differed in shape and stiffness to make the participants aware that tissues in the human body differ as well. Figure 1 shows the instructions provided to the participants before the pretest measurement was started.

### Study design

**Fig. 2:**
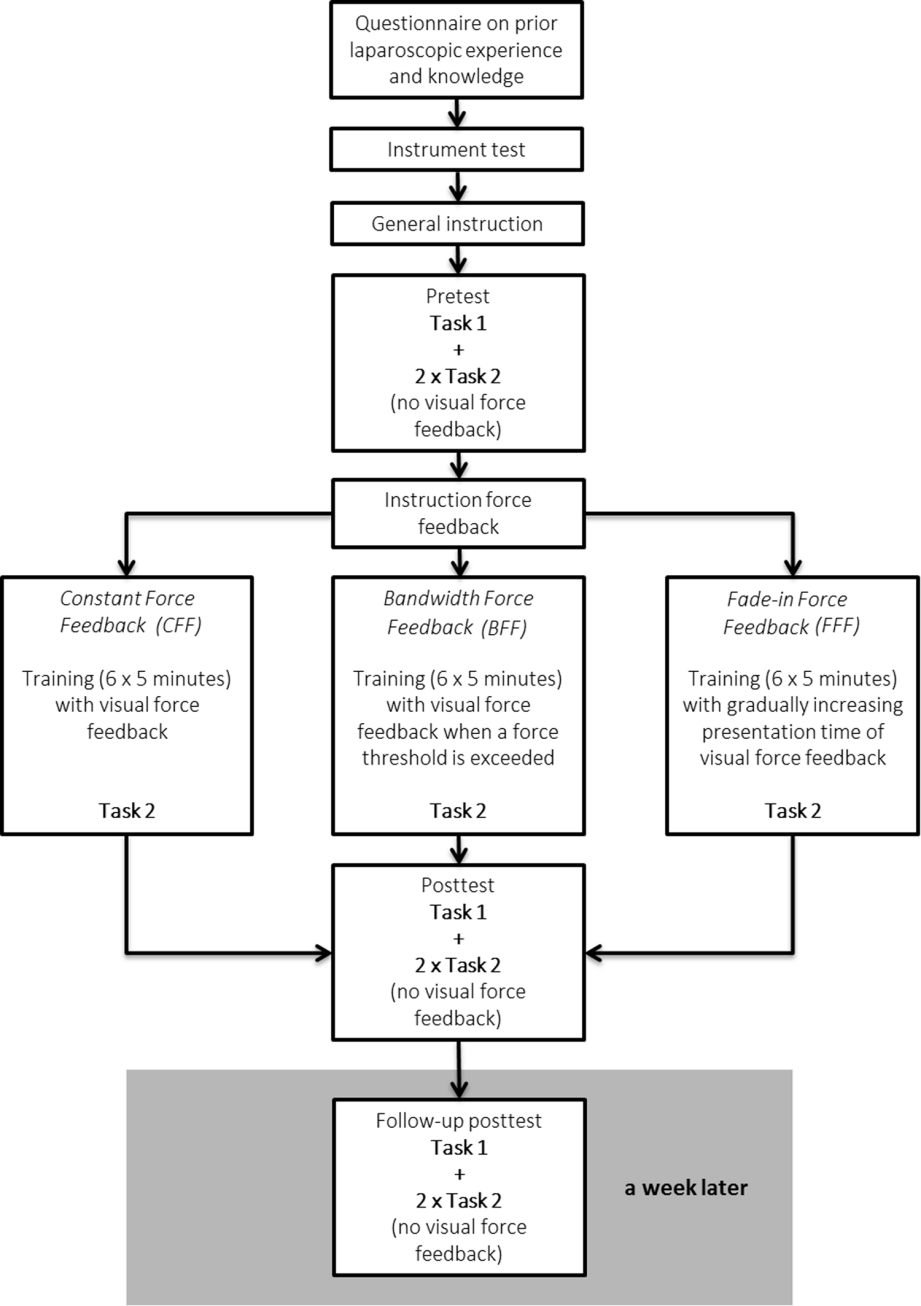
Schematic view of the study design

Participants performed the two different training tasks inside the ForMoST hybrid trainer. Task 1 was used in the pretest, post-test and follow-up test (Fig. 2). Task 2 was used in the pretest, training, post-test and follow-up test. Task 1 was used to observe whether the force feedback training with Task 2 generated transfers to Task 1 indicated by a decrease in force parameter outcomes values. The study consisted of two meetings: the duration of the first meeting was 90 min and the second meeting, scheduled 1 week later, had a duration of 15 min.

The training consisted of 6 trials of 5 min each. Participants received real-time visual force feedback during training according to the force feedback group assigned to. The CFF group received continuous feedback about their applied force. Participants in the BFF group were only presented with visual force feedback when their applied force exceeded the threshold of 5.3 N. The threshold was based on a previous study that defined the critical force level that causes tissue damage [19]. Once the visual force feedback was presented, it lingered for 10 s to give the participants the opportunity to notice the feedback and to correct their actions accordingly. The presented force feedback then disappeared again, but only if the exerted force was decreased below the threshold of 5.3 N. The FFF group was not exposed to force feedback in their first training trial. In the second training trial, participants were presented with force feedback solely in the first minute. The time force feedback was presented gradually increased every trail by a minute. In the last training trial, participants of the FFF group were continuously presented with force feedback.

### Feedback design

**Fig. 3:**
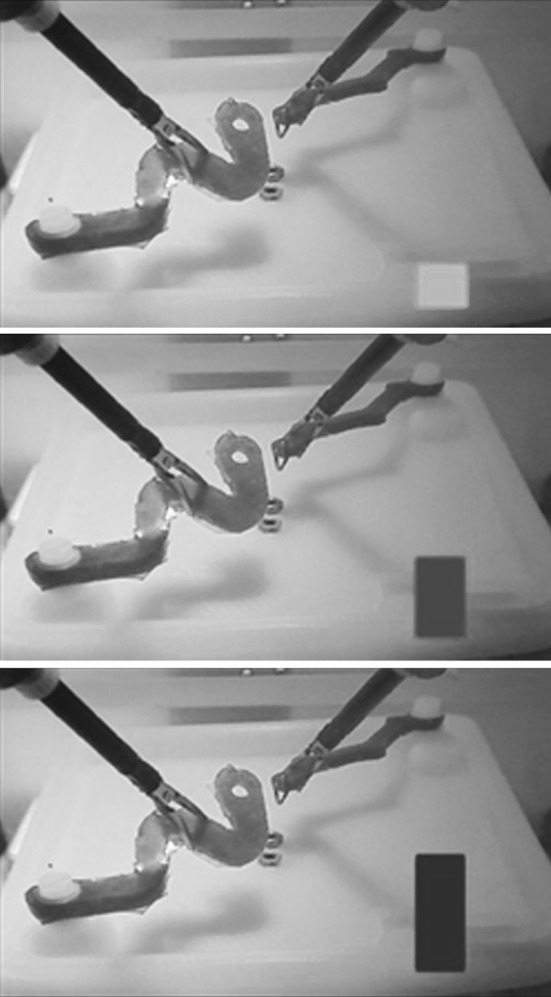
Display of Task 2 with force feedback during low (*green*), moderate (*orange*) and high (*red*) applied force (Color figure online)

To convey the force applied on the task, the visual force feedback design consisted of a vertical bar (Fig. 3) [16] that varied in size and colour as a result of the applied force on the task. A low amount of applied force was indicated by a small bar, and similarly a high amount of force exerted on the task was indicated by a larger bar. The colour of the force feedback bar was chosen consistent with existing preconceptions [16]. The bar gradually changed colour bottom-up from green to yellow to orange to red depending scaled with the amount of exerted force. Warning triangles were presented in each corner of the display if extreme force was applied to prevent rupture of the strips. Since the elasticity of the artificial tissue (silicon) is close to that of uterus tissue, the safety thresholds associated with uterus tissue were used in the colour scheme of the force feedback [19].

### Training protocol

First, participants signed an informed consent form and filled out a short demographics questionnaire. Next, participants familiarized themselves with the instruments, because understanding of equipment is important for safe laparoscopic surgery [20]. Prior to the pretest, the participants were presented with visual on-screen instructions how to complete Task 1 (Fig. 1). All participants were told to handle the tissues with care to prevent damage of the elastic components and to keep vision on the instruments at all time. After completing Task 1, instructions for Task 2 (Fig. 1) were presented on the display. Participants performed Task 2 twice to create a reliable baseline. All participants performed Task 1 (placement of thread in flap) and Task 2 (connection of the silicon strips) during the pretest without feedback of the tissue manipulation force.

Hereafter, all participants received instructions explaining the visual force feedback showed on the screen during training. As the type of force feedback during Task 2 was group dependent, this part of the explanation was different for each group. All participants were told that the training consisted of 6 trials of 5 min of Task 2. Participants were asked to complete Task 2 multiple times for the duration of each trial.

After the training, participants read the instructions for Task 1 again and were asked to perform the post-test (Task 1 and Task 2) without presentation of visual force feedback. A week later, all participants were asked to perform the follow-up test. The procedure was identical to the pretest and post-test. After completing Task 1 once and Task 2 twice, the participants received a certificate.

### Performance parameters

Based on the proven classification power in earlier studies [7], the parameters Maximum absolute force, Mean Absolute Nonzero Force, Force Volume and Max Force Area and Task (completion) time were selected to establish a learning effect and to differentiate between the groups that trained with different types of feedback [6, 7]

#### Mean Absolute Nonzero Force

The mean absolute force applied solely during application of force in Newton [6].

#### Maximum Absolute Force

The highest absolute force in Newton was applied on the training task during the measurement [6].

#### Force Volume

If the force data are presented in 3D, three orthogonal principal components can be found indicating the three largest standard deviations of the force. The Force Volume is the volume of an ellipsoid fitted around those three standard deviations [6].

#### Max Force Area

If the absolute force is presented in time, the Max Force Area indicates the largest surface area under the graph. A force area is created between the moment in time the absolute force becomes larger than zero and the following moment in time the absolute force becomes zero again. Max Force Area units are presented in Newton second and referred to as peak force in earlier research [6].

#### Task time

The time needed to complete the task, presented in seconds [6].

### Statistics

Task 2 is used to identify differences between CFF, BFF and FFF on learning efficiency. To ensure a valid pretest, post-test and follow-up test data of Task 2, the mean of two measurements was taken. A paired-sample t test was used to compare the pretest mean scores with the post-test mean scores of Task 1 and Task 2 separately. A paired-sample t test was also used to compare the pretest mean scores and follow-up test mean scores of Task 1 and Task 2 separately.

Differences between the mean scores of the three groups in the pretest, post-test and follow-up post-test of Task 2 were examined using multiple one-way MANOVA’s. Post hoc tests with Bonferroni correction were performed with a significance level of *p* < 0.05.

## Results

### Statistical differences between groups

The one-way MANOVA indicated no significant differences between the mean scores of the three groups in the pretest on Task 2. Although the one-way MANOVA of Task 2 on the post-test revealed no significant multivariate main effect between groups, a significant univariate main effect was observed for the Mean Absolute Nonzero Force (*F* (48, 2) = 4.303, *p* = .019, partial η2 = .152, power = .722) but not for the remaining force parameters. For this Absolute Nonzero force, the Bonferroni post hoc tests showed a significantly lower mean score for the CFF group compared to the BFF group (*p* = .005). The one-way MANOVA performed on the mean scores of the three groups in the follow-up test did not reveal any significant differences between groups.

### Differences between pre, post and follow-up measurements

#### Task 1

**Fig. 4:**
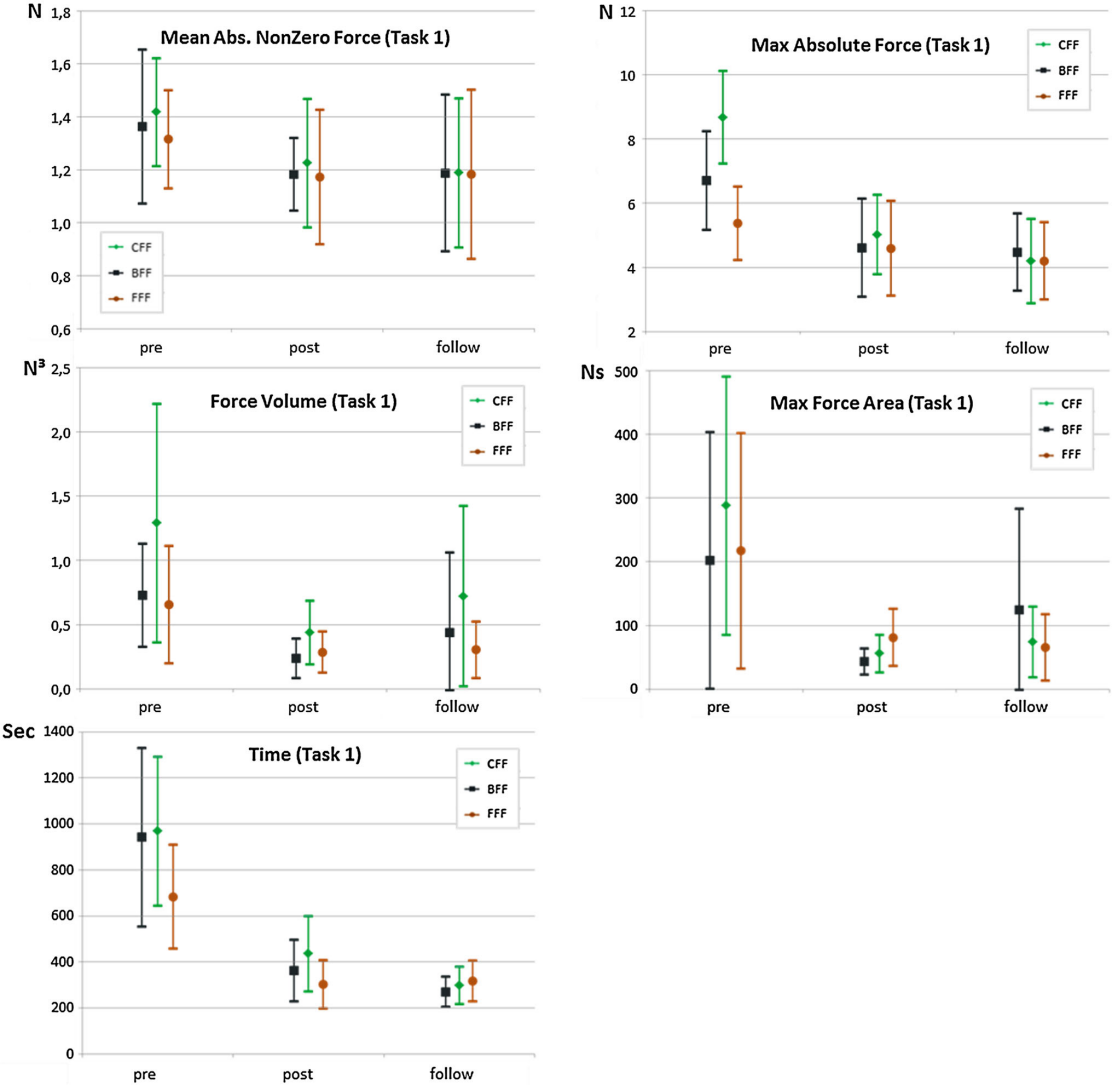
Mean scores for Mean Absolute Nonzero Force, Max Absolute Force, Force Volume, Max Force Volume and Time with 95 % confidence intervals of the untrained Task 1 divided for the three methods (*CFF* Constant Force Feedback, *BFF* Bandwidth Force Feedback, *FFF* Fade-in Force Feedback)

To get insight into the effect of the feedback type on the force and time parameter results, the parameter outcomes of the training trials in relation to the pre, post and follow-up trials are presented in Figs. 4 (Task 1) and 5 (Task 2).

Comparison of the pretest mean scores with the post-test mean scores with a paired sample t tests indicates that participants significantly decreased their applied Mean Absolute Nonzero Force *t*(48) = 2.441, *p* = .018; Max Absolute Force *t*(48) = 5.866, *p* < .001; Force Volume *t*(48) = 3.446, *p* = .001; Max Force Area *t*(48) = 3.419, *p* = .001; and Task time *t*(48) = 5.958, *p* < .001. A week after training, the participants in the follow-up test still applied significantly less Mean Absolute Nonzero Force *t*(48) = 2.02, *p* = .049; Max Absolute Force *t*(48) = 5.809, *p* < .001; Force Volume *t*(48) = 2.479, *p* = .017; Max Force Area *t*(48) = 2.692, *p* = .010; and Task time *t*(48) = 6.674, *p* < .001 compared with the pretest (Fig. 4).

#### Task 2

**Fig. 5:**
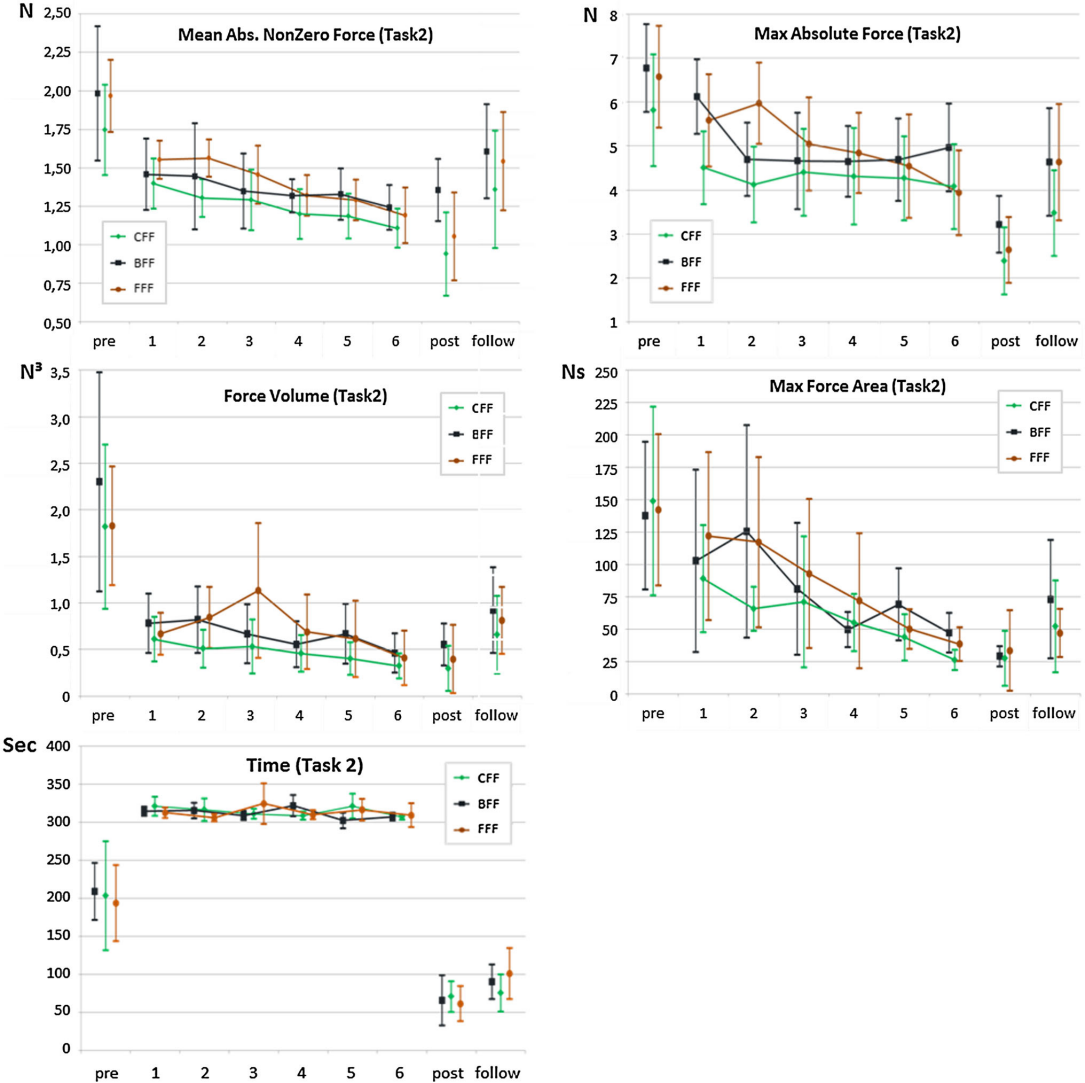
Mean scores for Mean Absolute Nonzero Force, Max Absolute Force, Force Volume, Max Force Volume and Time with 95 % confidence intervals of the trained Task 2, divided for the three methods (*CFF* Constant Force Feedback, *BFF* Bandwidth Force Feedback, *FFF* Fade-in Force Feedback)

The paired sample t tests indicated that the participants significantly decreased their applied force and Task time in the post-test compared to the pretest (Mean Absolute Nonzero Force *t*(49) = 6.656, *p* < .001; Max Absolute Force *t*(49) = 11.057, *p* < .001; Force Volume *t*(49) = 6.187, *p* < .001; Max Force Area *t*(49) = 6.153, *p* < .001; and Task time *t*(49) = 8.824, *p* < .001). Furthermore, when comparing the pretest mean scores to the follow-up test mean scores of Task 2, we find that the participants were able to significantly decrease their applied Mean Absolute Nonzero Force *t*(48) = .004, *p* = .004; Max Absolute Force *t*(48) = 5.321, *p* < .001; Force Volume *t*(48) = 4.633, *p* < .001; Max Force Area *t*(48) = 4.427, *p* < .001; and Task time *t*(48) = 7.221, *p* < .001 (Fig. 5).

## Discussion

The aim of this study was to determine whether different visual force feedback types (i.e. constant, bandwidth and fade-in) have different effects on the learning curve when acquiring tissue manipulation skills. Only the force parameter Mean Absolute Nonzero Force showed significantly lower mean scores for the BFF group compared with the other groups. This lack of meaningful differences between the groups in the follow-up test seems remarkable because of the difference in total time that participants received visual force feedback in the three groups.

Comparing the learning curve trajectories of the three groups provides insight into the impact of visual force feedback on the force parameters that reflect dangerous tissue handling (i.e. Max and mean NZ force and Force area). Participants in the FFF group applied relative high force in the first two trials, in comparison with the other groups. When the force feedback became more prevalent in the remaining trials, participants in the FFF group managed to improve their tissue manipulation skills in a faster rate until the level of the participants in the other groups was reached. This shows potential for more advanced tasks as it allows the trainee to decide to master basic skills (instrument handling, fulcrum effect, bimanual cooperation, etc.) first before focusing on tissue handling aspects.

Although all feedback types seem to work effectively for the performed tasks, the Bandwidth Force Feedback is the only type of feedback that respects individual progress. It therefore minimizes the duration of visual force feedback presentation while similar performance improvements are observed. This indicates that brief exposure to visual force feedback at the right moment in training is already sufficient to decrease the applied force.

Observing the results in general, one can clearly identify learning curves for all of the force parameters on the trained task. All participants significantly decreased their mean scores on all force parameters compared to the pretest. After 1 week, a clear training effect was still prevalent since participants performed the trained task with significantly lower mean scores on all of the force parameters compared to the pretest. Prospects of the training method are promising because laparoscopic tissue manipulation skills acquired in one and a half hour are still retained after a week. In addition, transfer to a different task with dissimilar characteristics is observed as well. Participants were able to significantly decrease their scores on all force parameters on a dissimilar untrained task. In the follow-up test, participants had significantly lower mean scores on the untrained task on aforementioned force parameters. The experimental training groups aside, one can conclude that the training method with visual force feedback is generally effective in decreasing the applied force.

### Limitations

Horeman et al. [9, 10] previously showed that participants significantly decreased their applied force when presented with constant visual force feedback compared to a control group where no feedback was given. This study aimed to tune the visual force feedback training method; therefore, the control group in this study was a group with constant feedback. The lack of a no visual force feedback group can be seen as a limitation of the current design.

Another limitation is the extended period of training on one task. Multiple participants reported to be bored as a result of the lengthy training trials. Usually, such emotional states can cause demotivation and decrease task engagement [21]. Ultimately, this could have resulted in a decreased potential to acquire the laparoscopic tissue manipulation skills.

Not using a power calculation to determine the required group size can be seen as a limitation. Instead, the study of Horeman et al. [10] was used to determine the absolute minimum group size required to distinguish the most important differences in performance. The maximum actual size was determined by the number of participants willing to collaborate.

### Recommendations

The study shows that training effects of the ForMoST device in combination with the presentation of visual force feedback are retained for at least a week. Second, these training effects also transfer to an untrained task with other characteristics. It is of utmost importance that the acquired laparoscopic skills can be transferred to the real occupational setting as well. Proving predictive validity would increase the legitimacy of this training method [22]. Further research is required to understand whether, and to what extent, the acquired laparoscopic skills are transferable to the OR. Reassessment on hybrid box trainers at a later point in time should also clarify the long-term retention of the acquired laparoscopic skills. Participants should be reassessed after an extended interval to reveal the effectiveness of the training method over time [23].

Of main importance for the student surgeons is to acquire laparoscopic tissue manipulation skills, which includes awareness of the consequences of too much applied tissue force and the level of their tissue interaction force. The training method that is used in this study supports the participant in acquiring those skills and should therefore be included in the laparoscopic surgical training curriculum. Adding requirements for force parameters scores in the performance assessment of residents will ensure surgeons possess better laparoscopic skills after completing training.

## Conclusion

All visual force feedback groups showed to be equally effective in decreasing participants applied task force. The learning curves recorded in training, the mean scores of the force parameters in post-test and the retention effects after a week indicate that training with visual force feedback results in enhanced laparoscopic tissue manipulation skills. As the Bandwidth Force Feedback type is only present when force levels are dangerous, it minimizes attentional distraction and is therefore preferable for training.

## References

[1] Blavier A (2006). Impact of 2D and 3D vision on performance of novice subjects using da Vinci robotic system. Acta Chir Belg.

[2] Spruit EN (2014). Optimal training design for procedural motor skills: a review and application to laparoscopic surgery. Psychol Res.

[3] Ahlberg G (2007). Proficiency-based virtual reality training significantly reduces the error rate for residents during their first 10 laparoscopic cholecystectomies. Am J Surg.

[4] Sinitsky DM, Fernando B, Berlingieri P (2012). Establishing a curriculum for the acquisition of laparoscopic psychomotor skills in the virtual reality environment. Am J Surg.

[5] Van den Dobbelsteen JJ, Schooleman A, Dankelman J (2007). Friction dynamics of trocars. Surg Endosc.

[6] Horeman T (2014). Force-based assessment of tissue handling skills.

[7] Horeman T (2014). Assessment of laparoscopic skills based on force and motion parameters. Biomed Eng IEEE Trans Haptics.

[8] Martin JA (1997). Objective structured assessment of technical skill (OSATS) for surgical residents. Br J Surg.

[9] Horeman T (2014). Visual force feedback improves knot-tying security. J Surg Educ.

[10] Horeman T (2014). Learning from visual force feedback in box trainers: tissue manipulation in laparoscopic surgery. Surg Endosc.

[11] Buchanan JJ, Wang C (2012). Overcoming the guidance effect in motor skill learning: feedback all the time can be beneficial. Exp Brain Res.

[12] Lam CF (2011). The impact of feedback frequency on learning and task performance: Challenging the “more is better” assumption. Org Behav Hum Decis Process.

[13] Magill RA (2007). Motor learning and control, concepts and applications.

[14] Patterson Jae T, Carter Michael J, Hansen Steve (2013). Self-controlled KR schedules: does repetition order matter?. Hum Mov Sci.

[15] Wulf G (2007) Attention and motor skill learning. Human Kinetics, Champaign, IL

[16] Sigrist R (2013). Augmented visual, auditory, haptic, and multimodal feedback in motor learning: a review. Psychon Bull Rev.

[17] Ribeiro DC (2011). Extrinsic feedback and management of low back pain: a critical review of the literature. Man Ther.

[18] Horeman T (2012). Visual force feedback in laparoscopic training. Surg Endosc.

[19] Rodrigues SP (2012). Suturing intraabdominal organs: when do we cause tissue damage?. Surg Endosc.

[20] van Hove PD (2012). Effect of basic laparoscopic skills courses on essential knowledge of equipment. Surg Innov.

[21] Fisher CD (1993). Boredom at work: a neglected concept. Hum Relat.

[22] Mané AM, Adams JA, Donchin E (1989). Adaptive and part-whole training in the acquisition of a complex perceptual-motor skill. Acta Psychol.

[23] Hiemstra E (2012) Acquiring minimally invasive surgical skills. Department of Minimally Invasive Surgery in Gynaecology, Faculty of Medicine/Leiden University Medical Center (LUMC), Leiden University

